# Toxicity of anti-carcinogenic retinoids in organ culture.

**DOI:** 10.1038/bjc.1977.11

**Published:** 1977-01

**Authors:** D. R. Bard, I. Lasnitzki

## Abstract

**Images:**


Br. J. Cancer (1977) 35, 115.

### Short Communication

### TOXICITY OF ANTI-CARCINOGENIC RETINOIDS IN ORGAN CULTURE

D. R. BARD AND I. LASNITZKI

*From the Strangeways Research Laboratory, Wort's Causeway, Cambridge, CB1 4RN.*

Received 23 July 1976 Accepted 3 August 1976

CONSIDERABLE interest has been aroused in recent years by the ability of retinol, retinoic acid and some of their derivatives to oppose chemically induced carcinogenesis of various epithelial tissues in experimental animals (Chu and Malmgren, 1965; Saffiotti *et al.*, 1967; Bollag, 1972, 1974, 1975). These compounds also inhibit and reverse the changes induced by 3-methylcholanthrene in organ cultures of mouse prostate (Lasnitzki, 1955; Lasnitzki and Goodman, 1974), whilst clinical trials show that retinoic acid causes the regression of bladder papillomas and keratinizing skin tumours (Bollag and Ott, 1970; Evard and Bollag, 1972). The usefulness of vitamin A in the chemotherapy of tumours is, however, limited by the toxicity of the doses required to inhibit carcinogenesis, and the search for more suitable compounds has centred upon the synthesis of analogues of retinol or retinoic acid, in an attempt to produce efficient anti-tumour agents of low toxicity.

The toxicity of retinoids can largely be attributed to their action, at higher than physiological concentrations, in destabilizing membranes, thus causing the release of lysosomal enzymes (Dingle and Fell, 1963). This property is most readily observed in cartilage, in which these enzymes degrade the matrix, with the resultant loss of protein-polysaccharides. Goodman *et al.* (1974) have followed the effect of some retinoids by metachromatic staining and by measuring the appearance of proteoglycan fragments in the medium of organ cultures of rabbit ear cartilage.

In the present study, the release of sulphate from the matrix of rabbit ear cartilage has been used as a measure of toxicity. The protein-polysaccharides of rabbit ear cartilage were labelled with $^{35}SO_4^{2-}$ in organ culture, and the loss of the isotope in the presence of 13 retinoids determined, and related to their structure. The loss of metachromasia was also examined.

Cartilage was explanted by a modified Trowell technique, and incubated for 20 h with Medium 199 (Morgan, Morton and Parker, 1950) containing 15% foetal calf serum and 2$\mu$Ci/ml [$^{35}$S]sodium sulphate. After this initial incubation, unbound sulphate was removed from the cartilage by thorough washing, and the cultures carried on, for up to 5 days, in the presence of retinol, retinoic acid, or one of their derivatives, in a concentration of 2, 5, 10 or 20 $\mu$M. The structures of the synthetic retinoids are shown in Fig. 1. At the completion of the incubation, the labelled cartilage was completely digested in protease, and the radioactivity of the digest and the medium measured by liquid scintillation counting. Sulphate release, even in the presence of the most active compounds, was linear for the first 3 days of incubation, and results were expressed as the proportion of sulphate released during this time, divided by the values for

FIG. 1.—Structures of synthetic vitamin A analogues.

the retinoid-free controls for the same set of incubations.

Explants which had not been exposed to the isotope were maintained under identical conditions to the labelled tissue, for periods of up to 7 days, for examination of their metachromasia. These explants were fixed, processed for histology and stained with toluidine blue.

Retinoic acid, and all its analogues which retained the carboxylic acid group, induced the release of 60% to 90% of the labelled sulphate from the explants during the first 3 days of incubation, a value over twice that of the controls. None of these acidic compounds were significantly different in their effects from retinoic acid, although this category included structures as diverse as the dimethylacetylcyclopentenyl (DACP: Ro8-7699) and trimethylmethoxyphenyl (TMMP: Ro10-1670) analogues, all-trans-$A_2$-retinoic acid (Ro8-7057), 13-cis-$\beta$-retinoic acid (Ro4-3780) and 13-cis-$\alpha$-retinoic acid (Ro8-7201) (Fig. 2). Retinol, and derivatives of retinoic acid in which the carboxyl group had been replaced by a less polar moiety, such as an ether, ester or amide, caused much less sulphate to be lost, and 2 of these compounds, Ro10-9359 and Ro1-5218, were significantly less active than retinol ($P = 0{\cdot}02$) (Fig. 2.) The efficiency of retinoic acid and the DACP derivative, Ro8-7699, and the lower activity of retinol in causing the breakdown of cartilage has been noted by Goodman and co-workers (1974). The concentrations of the vitamin A compounds had little influence on their activity within the range used, and none of the 5 compounds examined showed any significant difference between the 2-, 10-, and 20-$\mu$M concentrations (Fig. 3).

The histological changes in the cartilage supported the results of the sulphate release experiments. Tissue kept for 7 days without retinoids showed small losses of metachromasia from the edges of the explants (Fig. 4a), whereas cartilage maintained in the presence of 5$\mu$M retinoic acid and the other acidic derivatives, such as

FIG. 2.—Effects of vitamin A analogues on the release of $^{35}SO_4^{2-}$ from pre-labelled rabbit ear cartilage; concentration of each compound = 10 $\mu$M.

FIG. 3.—Effects of different concentrations of retinoic acid, retinol, 11–1430, 1–5218 and 10–1670 on the release of sulphate from pre-labelled rabbit ear cartilage.

Ro10-1670, showed a total lack of staining, and signs of cell shrinkage with vacuolation of the cytoplasm (Fig. 4*c*, *d*). The same concentration of retinol caused only a partial depletion of metachromasia throughout the matrix, and better cell survival (Fig. 4*b*), while explants incubated with the ethyl ester of the TMMP derivative of retinoic acid, Ro10-9359, retained almost as much metachromasia as the controls, and losses were restricted to the edges of the explants (Fig. 4*e*).

FIG. 4.—Effects of vitamin A analogues on the loss of metachromasia from rabbit ear cartilage after 7 days' culture; (a) Control (without retinoid), (b) Retinol, (c) Retinoic acid, (d) 10-1670, (e) 10-9359. Concentration = 5$\mu$M. Toluidene blue. × 150.

The results support the hypothesis that retinoic acid may act as a detergent in destabilizing lysosomal membranes (Dingle and Fell, 1963), since all the compounds with carboxylic acid groups are active, and the replacement of the acid moiety by less hydrophilic groups would diminish their efficiency as surfactants. The results also imply that in cartilage, at least, ethers, esters, and amides are not converted extensively to retinoic acid. The possibility that these conversions may take place in other tissues, and consequently affect the pharmacology of these compounds, cannot be excluded at present.

The anti-carcinogenic activity appears to be unrelated to the interaction with membranes, since the most effective anti-carcinogens are not necessarily the most polar (Lasnitzki, 1976), and detergents of different structures do not oppose tumour development (Lasnitzki and Goodman, 1974). The identification of compounds which combine potent anti-carcinogenic action with low activity in destabilizing lysosomes offers considerable hope for the eventual development of an effective anti-tumour agent of low general toxicity.

We are greatly indebted to Dr W. Bollag and Dr N. I. Pollitt of Hoffmann–La Roche, Basle, Switzerland, and Welwyn Garden City, England, for the generous gift of the vitamin A analogues.

We thank Miss A. A. Turner for skilled technical assistance. The work was supported by the Cancer Research Campaign.

### REFERENCES

BOLLAG, W. (1972) Prophylaxis of Chemically Induced Benign and Malignant Epithelial Tumours by Vitamin A Acid (Retinoic Acid). *Eur. J. Cancer*, **8**, 689.

Bollag, W. (1974) Therapeutic Effect of an Aromatic Retinoic Acid Analog on Chemically Induced Skin Papillomas and Carcinomas in Mice. *Eur. J. Cancer*, **10**, 731.

Bollag, W. (1975) Therapy of Epithelial Tumours with an Aromatic Retinoic Acid Analog. *Chemotherapy*, **21**, 236.

Bollag, W. & Ott, F. (1970) Retinoic Acid. Topical Treatment of Senile or Actinic Keratosis and Basal Cell Carcinomas. *Agents Actions*, **1**, 172.

Chu, E. W. & Malmgren, R. A. (1965) An Inhibitory Effect of Vitamin A on the Induction of Tumours of Forestomach and Cervix in the Syrian Hamster by Carcinogenic Polycyclic Hydrocarbons. *Cancer Res.*, **25**, 884.

Dingle, J. T. & Fell, H. B. (1963) Studies on the Mode of Action of Excess Vitamin A, 6, Lysosomal Protease and the Degradation of Cartilage Matrix. *Biochem. J.*, **87**, 403.

Evard, J. P. & Bollag, W. (1972) Konservative Behandlung der rezidivierenden Harnblasenpapillomatose mit Vitamin A Saüre. *Schweiz. Med. Wschr.*, **102**, 1880.

Goodman, D. S., Smith, J. E., Hembry, R. M. & Dingle, J. T. (1974) Comparison of the Effects of Vitamin A and its Analogs upon Rabbit Ear Cartilage in Organ Culture and upon Growth of the Vitamin A Deficient Rat. *J. Lipid Res.*, **15**, 406.

Lasnitzki, I. (1955) The Influence of A-hypervitaminosis on the Effect of 20-methylcholanthrene on Mouse Prostate Glands *in vitro*. *Br. J. Cancer*, **9**, 434.

Lasnitzki, I. (1976) Reversal of Methylcholanthrene-induced Changes in Mouse Prostates *in vitro* by Retinoic Acid and its Analogues. *Br. J. Cancer*, **34**, 239.

Lasnitzki, I. & Goodman, D. S. (1974) Inhibition of the Effects of Methylcholanthrene on Mouse Prostate in Organ Culture by Vitamin A and its Analogs. *Cancer Res.*, **34**, 1564.

Morgan, J. F., Morton, H. J. & Parker, R. C. (1950) Nutrition of Animal Cells in Organ Culture. *Proc. Soc. exp. Biol. Med.*, **73**, 1.

Saffiotti, U., Montesano, R., Sellakumar, A. R. & Borg, S. A. (1967) Experimental Cancer of the Lung. Inhibition by Vitamin A of the Induction of Tracheol Broncheal Squamous Metaplasia and Squamous Cell Tumours. *Cancer, N. Y.*, **20**, 857.


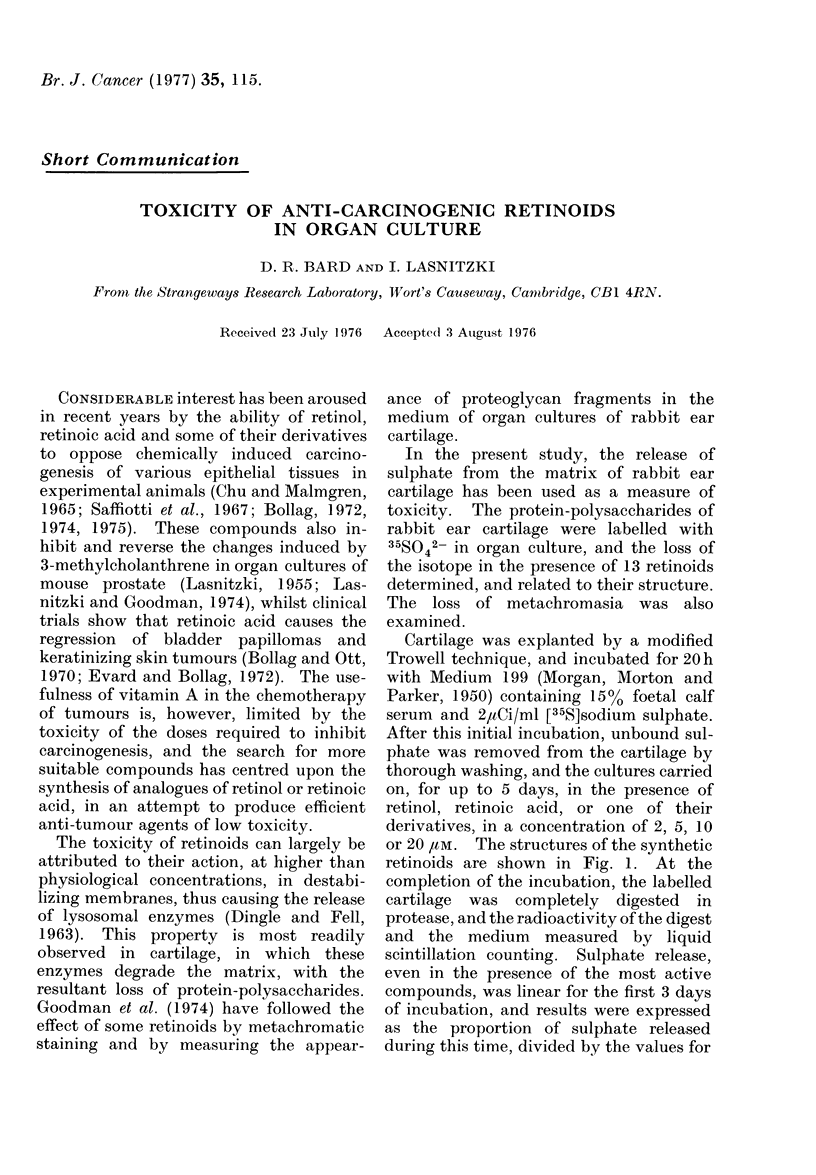

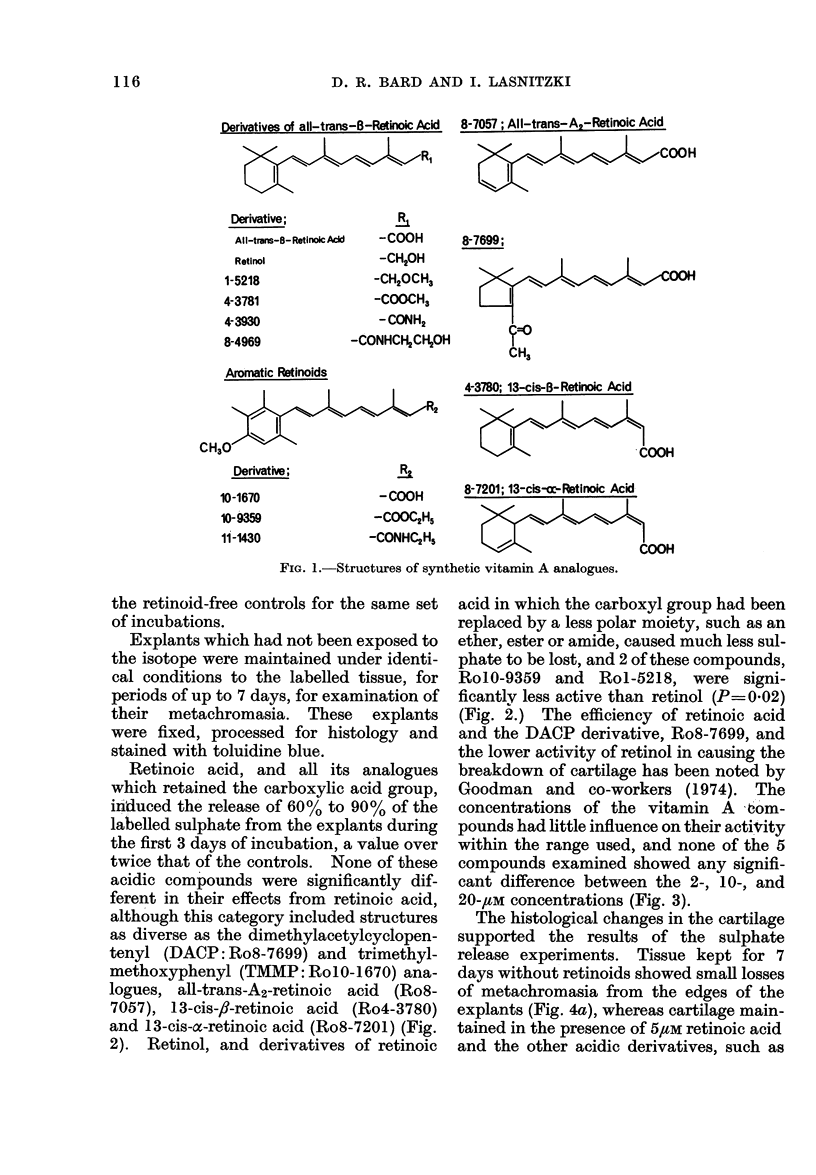

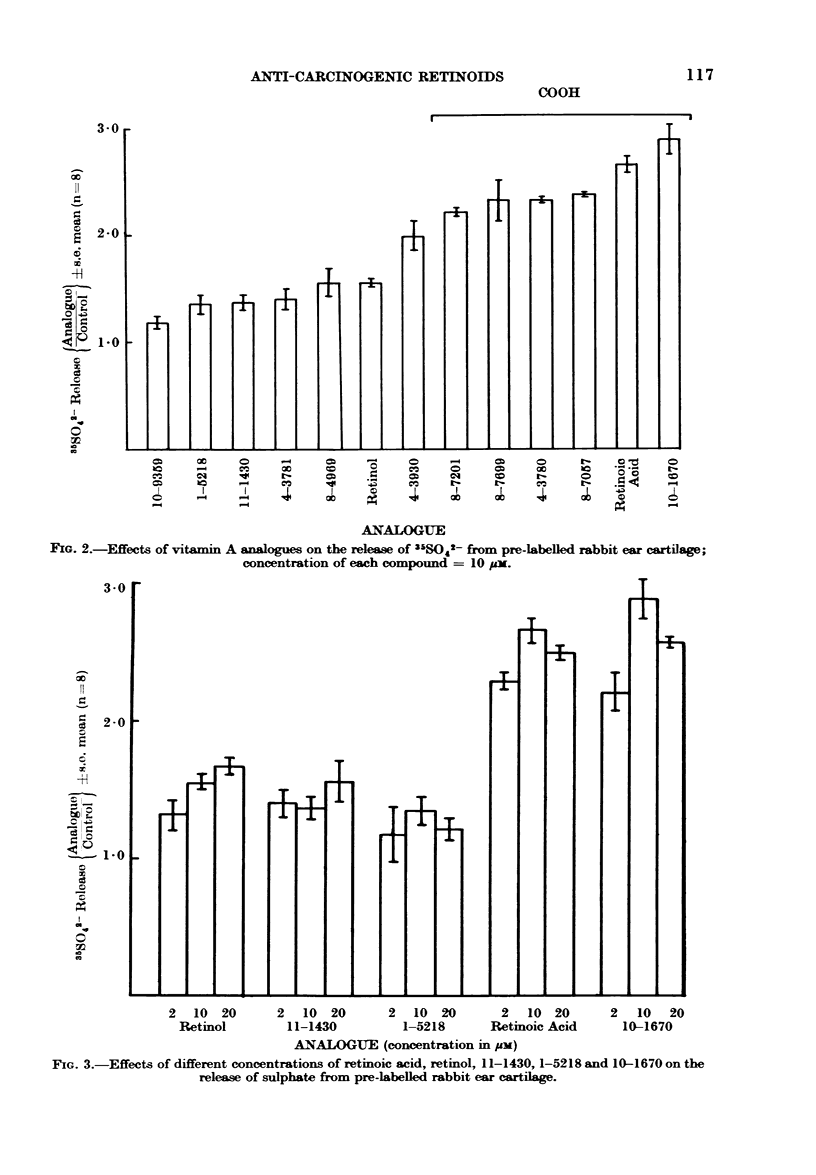

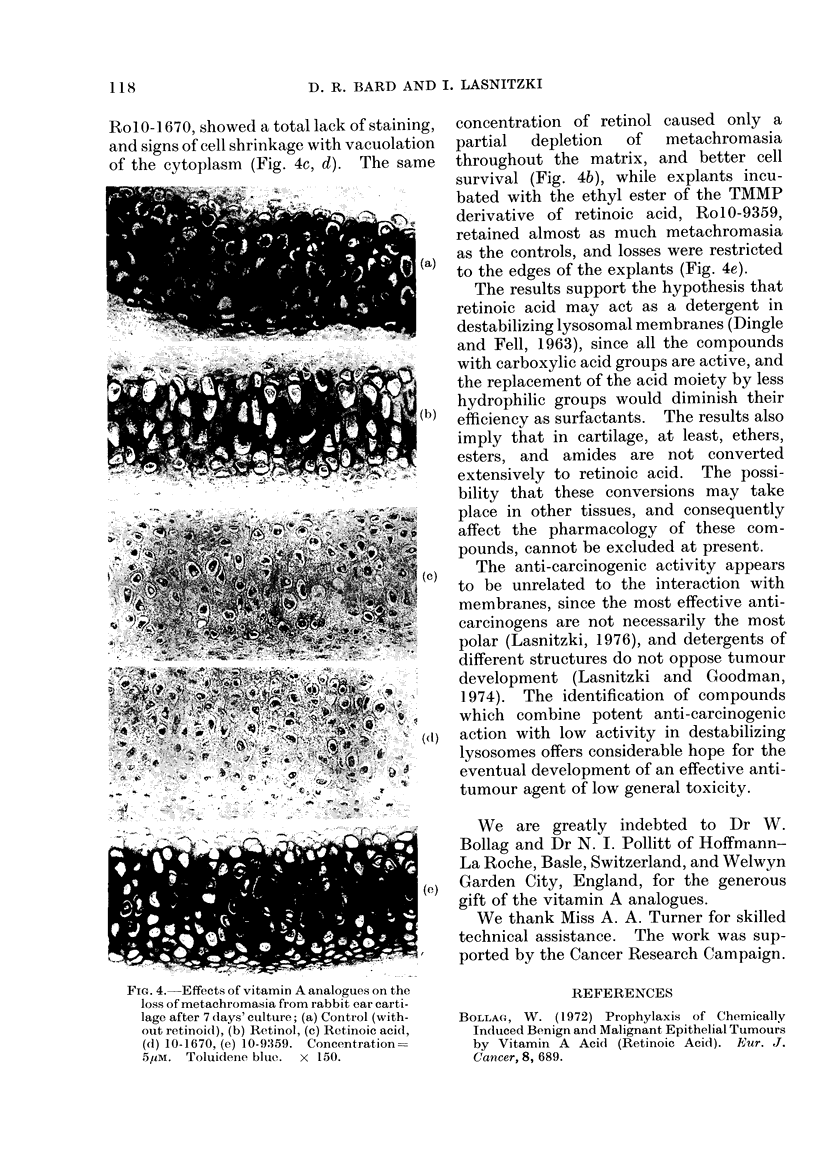

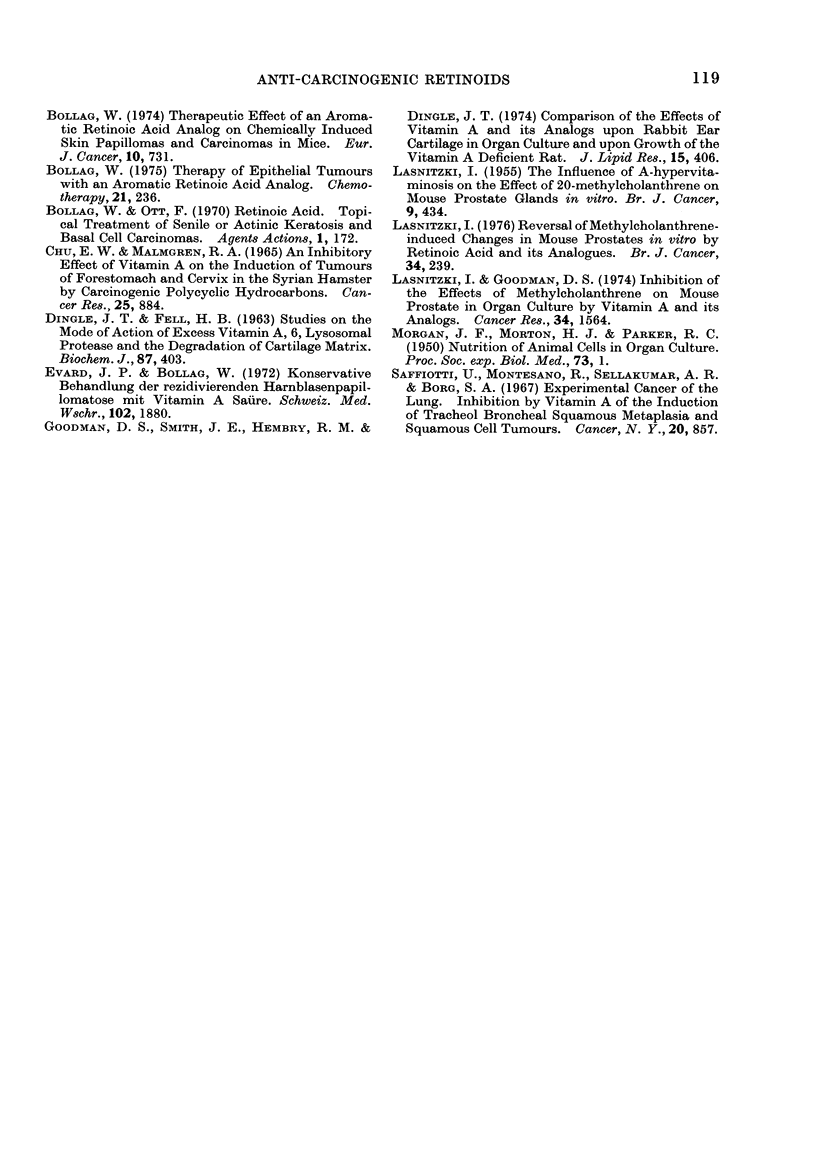


## References

[OCR_00383] Bollag W., Ott F. (1970). Retinoic acid: topical treatment of senile or actinic keratoses and basal cell carcinomas.. Agents Actions.

[OCR_00364] Bollag W. (1972). Prophylaxis of chemically induced benign and malignant epithelial tumors by vitamin A acid (retinoic acid).. Eur J Cancer.

[OCR_00372] Bollag W. (1974). Therapeutic effects of an aromatic retinoic acid analog on chemically induced skin papillomas and carcinomas of mice.. Eur J Cancer.

[OCR_00378] Bollag W. (1975). Therapy of epithelial tumors with an aromatic retinoic acid analog.. Chemotherapy.

[OCR_00388] Chu E. W., Malmgren R. A. (1965). An inhibitory effect of vitamin A on the induction of tumors of forestomach and cervix in the Syrian hamster by carcinogenic polycyclic hydrocarbons.. Cancer Res.

[OCR_00401] Evard J. P., Bollag W. (1972). Konservative Behandlung der rezidivierenden Harnblasenpapillomatose mit Vitamin-A-Säure. Vorläufige Mitteilung.. Schweiz Med Wochenschr.

[OCR_00395] FELL H. B., DINGLE J. T. (1963). Studies on the mode of action of excess of vitamin A. 6. Lysosomal protease and the degradation of cartilage matrix.. Biochem J.

[OCR_00407] Goodman D. S., Smith J. E., Hembry R. M., Dingle J. T. (1974). Comparison of the effects of vitamin A and its analogs upon rabbit ear cartilage in organ culture and upon growth of the vitamin A-deficient rat.. J Lipid Res.

[OCR_00413] LASNITZKI I. (1955). The influence of A hypervitaminosis on the effect of 20-methylcholanthrene on mouse prostate glands grown in vitro.. Br J Cancer.

[OCR_00425] Lasnitzki I., Goodman D. S. (1974). Inhibition of the effects of methylcholanthrene on mouse prostate in organ culture by vitamin A and its analogs.. Cancer Res.

[OCR_00419] Lasnitzki I. (1976). Reversal of methylcholanthrene-induced changes in mouse prostates in vitro by retinoic acid and its analogues.. Br J Cancer.

[OCR_00431] MORGAN J. F., MORTON H. J., PARKER R. C. (1950). Nutrition of animal cells in tissue culture; initial studies on a synthetic medium.. Proc Soc Exp Biol Med.

[OCR_00436] Saffiotti U., Montesano R., Sellakumar A. R., Borg S. A. (1967). Experimental cancer of the lung. Inhibition by vitamin A of the induction of tracheobronchial squamous metaplasia and squamous cell tumors.. Cancer.

